# Semaphorin 3A regulates alveolar bone remodeling on orthodontic tooth movement

**DOI:** 10.1038/s41598-022-13217-x

**Published:** 2022-06-02

**Authors:** Hirokazu Kamei, Takenobu Ishii, Yasushi Nishii

**Affiliations:** Department of Orthodontics, Tokyo Dental College, Tokyo, Japan

**Keywords:** Biochemistry, Cell biology, Endocrinology

## Abstract

Semaphorin 3A (Sema3A) promotes osteoblast differentiation and inhibits osteoclast differentiation. In the present study, we observed the regulation of alveolar bone remodeling by Sema3A during orthodontic tooth movement (OTM). Four inflammatory cytokines (IL-1β, IL-6, TNFα, and INF-γ) involved in OTM were applied to osteoblasts in vitro, and *Sema3A* expression was determined by reverse-transcription quantitative polymerase chain reaction (RT-qPCR). In vivo, springs were attached to the maxillary first molars of C56BL/6J mice (OTM model) and the localization of Sema3A was confirmed by immunofluorescent. Recombinant Sema3A (rSema3A) was locally injected into the OTM model. Inflammatory cytokine localization in the OTM model was confirmed by immunohistochemistry. In vivo, more Sema3A was observed on the tension side in the OTM group. Injection of rSema3A into the OTM model increased mineralization on the tension side and decreased the number of osteoclasts on the compression side. In vitro, IL-1β significantly increased *Sema3A* mRNA levels. Immunohistochemistry for IL-1β in vivo showed more concentrated staining in the periodontal ligament on the tension side than on the compression side. In summary, our findings revealed the distribution of Sema3A in the periodontal ligament and demonstrated that rSema3A administration promotes bone formation and inhibits bone resorption during OTM.

## Introduction

Bones are dynamic organs with constant bone resorption by osteoclasts and remodeling by bone mineralization by osteoblasts. Orthodontic tooth movement (OTM) results in alveolar bone remodeling in response to mechanical loading, bone resorption of osteoclasts on the compression side, and bone formation of osteoblasts on the tension side. Nuclear factor kappa B (RANK), RANK ligand (RANKL), and osteoprotegerin (OPG) are thought to be important proteins that control osteoclast function in OTM^1,2^. Various cytokines and proteins are involved in the process of OTM^3–5^. Although it is known that IL-10 inhibit osteoclastogenesis by boosting OPG and reducing RANKL production by osteoblasts on tension side^6^, there are few studies show how bone formation is promoted.

A recent study revealed that Semaphorin 3A (Sema3A) exerts an osteoprotective effect by both suppressing bone resorption and increasing bone formation^7^. Semaphorins are a family of cell-surface and soluble proteins that regulate cell–cell interactions as well as cell differentiation, morphology, and function. The common and defining feature of the semaphorin family is the presence of the extracellular SEMA domain and the cysteine-rich plexin, semaphorin, and integrin domain (PSI domain), which are both at the amino terminal end of the protein. The semaphorin family includes 8 subclasses: classes 1–7 and V^8,9^.

Sema3A is a secreted class 3 protein that is mainly secreted by osteoblasts, which inhibits the migration of osteoclast progenitors by suppressing the activation of Ras homolog family member A (RhoA). In addition, Plexin A1 promotes osteoclastogenesis by activating immune-receptor tyrosine-based activation motif (ITAM) signaling through the formation of the Plexin A1- triggering receptor expressed on myeloid cells 2 (TREM2)—DNAX-activating protein of 12 kDa (DAP12) complex^10^. Binding of Sema3A to neuropilin-1 inhibits the differentiation potential of osteoclast progenitors by drawing Plexin-A1 away from TREM2. Moreover, Sema3A promotes osteoblast differentiation by activating the canonical Wnt/β-catenin pathway^11^. Sema3A is thought to play a crucial role in the bone formation phase, in which osteoblasts extensively produce bone; at the same time, it restrains osteoclasts from migrating to the formation sites and resorbing the newly formed bone^7^. Sema3A administration decreased bone loss after ovariectomy by inhibiting osteoclastic bone resorption and promoting osteoblastic bone formation^7^. Sema3A is also involved in the establishment of a mouse tooth eruption pathway by modulating osteoclast activity^12^.

Although it has been shown that Sema3A is produced in human periodontal ligaments (PDL), such as by pre-osteoblasts and PDL fibroblasts^13^, it is not known whether it is involved in OTM. Although mechanical stress and some inflammatory cytokines stimulate periodontal ligament cells to regulate RANKL expression^14–16^, few is known about the factors that regulate Sema3A expression.

Using an OTM model, we showed how Sema3A affects OTM and identified factors influencing *Sema3A* expression in vivo and in vitro. These results help us explain one of the mechanisms of OTM and bone remodeling.

## Results

### Number of osteoclasts and *Sema3A* expression after OTM

**Figure 1:**
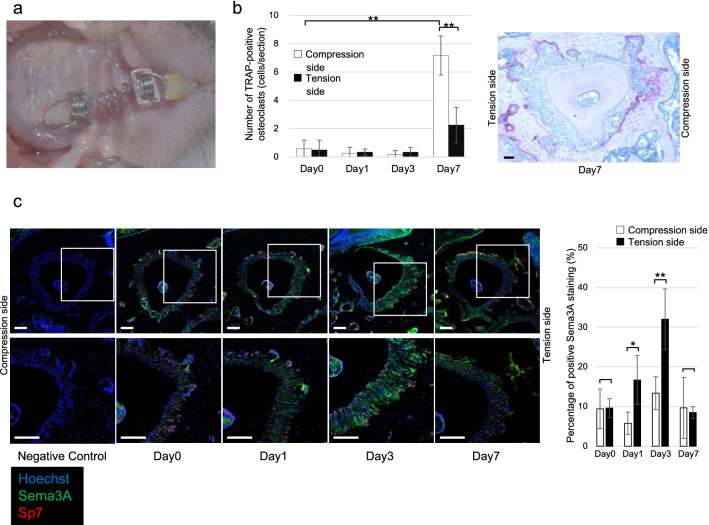
(**a**) Experimental model of orthodontic tooth movement (OTM). (**b**) Number of osteoclasts in the first molar palatal root region on compression and tension sides after OTM (n = 4) and TRAP staining of day 7. The number of osteoclasts was significantly increased on the compression side on day 7 and also a significant increase on the compression side compared to the tension side on day 7. Data are presented as the mean ± SD. *p < 0.05; **p < 0.01. Scale bars = 50 µm. (**c**) Immunofluorescent staining of Sema3A and Sp7 in the palatal roots. On day 1 and 3, more Sema3A was observed on the tension side than on the compression side (n = 5). Blue, Hoechst; Green, Sema3A; Red, Sp7. Data are presented as the mean ± SD. *p < 0.05; **p < 0.01. Scale bars = 100 µm.

TRAP staining was performed on tissue sections to observe osteoclasts on the compression and tension sides of mice after OTM. There was no significant difference in the number of osteoclasts between the compression and tension sides on day 1 (compression: 0.25 ± 0.43 cells/section, p = 0.999; tension: 0.33 ± 0.24 cells/section, p = 1.000) or day 3 (compression: 0.17 ± 0.29 cells/section, p = 0.997; tension: 0.34 ± 0.34 cells/section, p = 1.000) compared with day 0 (compression: 0.58 ± 0.59 cells/section, tension: 0.50 ± 0.69 cells/section). However, the number of osteoclasts was significantly increased on the compression side on day 7 (compression: 7.17 ± 1.39 cells/section, p = 0.000; tension: 2.25 ± 1.23 cells/section, p = 0.144). There was also a significant increase on the compression side compared to the tension side on day 7 (p = 0.000) (Fig. 1b).

Immunofluorescent staining revealed the distribution of Sema3A during OTM. No significant difference in Sema3A secretion was observed between the compression and tension sides on day 0 (compression: 9.43 ± 5.58%; tension: 9.64 ± 2.66%, p = 0.940) and day7 (compression: 9.67 ± 8.59%; tension: 8.50 ± 1.65%, p = 0.773), but more Sema3A was observed on the tension side than on the compression side on day 1 (compression: 5.80 ± 3.12%; tension: 16.70 ± 6.85%, p = 0.012) and day 3 (compression: 13.40 ± 4.62%; tension: 32.01 ± 8.54%, p = 0.003). In addition, Sema3A was observed especially around Sp7-positive cells on day 3 (Fig. 1c). These results indicate that Sema3A is secreted by osteoblast in the early phase of OTM.

### ALP activity and mineralized nodule formation by osteoblasts

It has been suggested that osteoblasts in the jaw have different properties from those of osteoblasts from other bone^17–19^. To confirm the effect of rSema3A on osteoblasts derived from the maxilla of the mouse, ALP and alizarin red staining was performed.

**Figure 2:**
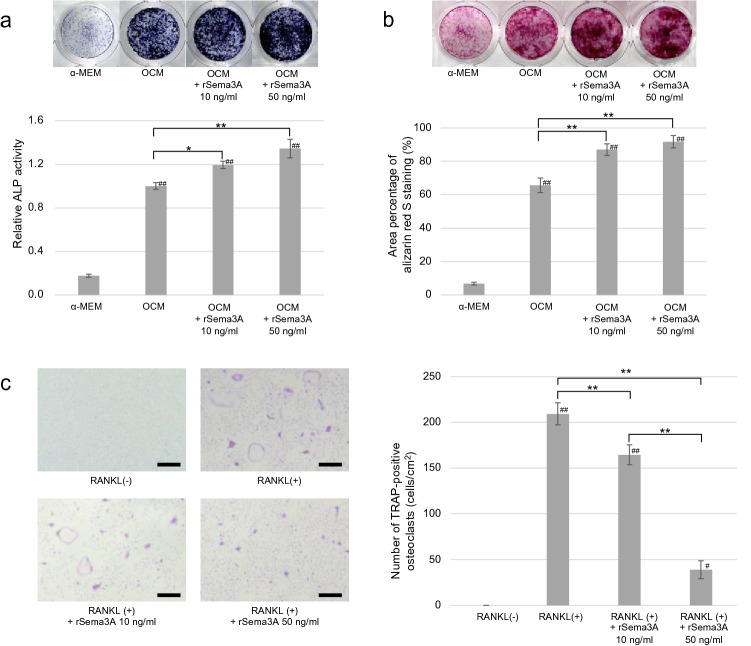
Osteoblasts were cultured in α-MEM and OCM with or without rSema3A (10, 50 ng/mL). (**a**) Alkaline phosphatase (ALP) staining and relative ALP activity (n = 3) at day 7. 10 ng/mL and 50 ng/mL rSema3A was significantly higher than that of the control cells. (**b**) Alizarin red S staining and area percentage of alizarin red S staining (n = 3) at day 21. 10 ng/mL and 50 ng/mL rSema3A significantly enhanced osteoblast extracellular calcification compared to control cells. *p < 0.05 and **p < 0.01. ^#^*p* < 0.05 and ^##^*p* < 0.01 vs. α-MEM (**c**) TRAP staining and number of TRAP-positive osteoclasts (n = 3). Bone marrow-derived monocyte/macrophage precursor cells (BMMs) were cultured with or without rSema3A (10, 50 ng/mL). rSema3A significantly reduced osteoclast formation. *p < 0.05 and **p < 0.01. ^#^*p* < 0.05, ^##^*p* < 0.01 vs. RANKL (-). Scale bars = 500 µm. Data are presented as mean ± SD.

In ALP staining, cells treated with 10 ng/mL and 50 ng/mL rSema3A were stained stronger than the control, and the relative ALP activity of cells treated with 10 ng/mL (1.19 ± 0.03, p = 0.017) and 50 ng/mL (1.34 ± 0.08, p = 0.000) rSema3A was significantly higher than that of the control cells (1 ± 0.03), with 50 ng/mL rSema3A having the largest effect (Fig. 2a).

Treatment with 10 ng/mL (87.0 ± 3.5%, p = 0.001) and 50 ng/mL (91.8 ± 3.7%, p = 0.000) rSema3A significantly enhanced osteoblast extracellular calcification compared to control cells (65.6 ± 4.4%) (Fig. 2b).

### TRAP staining of BMMs

TRAP staining of BMMs showed significantly reduced osteoclast formation both with 10 ng/mL (164.5 ± 10.9 cells/cm^2^, p = 0.006) and 50 ng/mL (38.9 ± 9.8 cells/cm^2^, p = 0.000) rSema3A groups compared to the control cells (209.3 ± 12.0 cells/cm^2^) (Fig. 2c).

### Injection of rSema3A in mice

**Figure 3:**
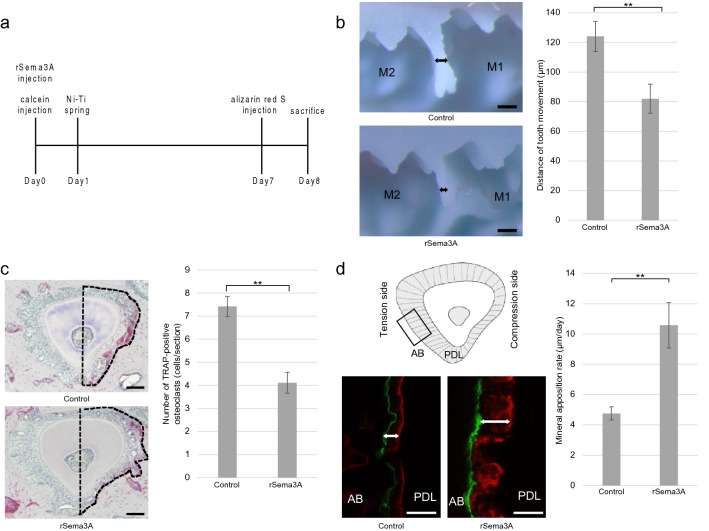
(**a**) Schematic diagram of rSema3A, calcein, and alizarin red S injection time course. rSema3A and calcein were injected 1 day before Ni–Ti coil spring attachment. Alizarin red S was injected 1 day before sacrifice. (**b**) Distance of tooth movement. Black arrows on the impression show orthodontic tooth movement (OTM) distance of the rSema3A injection group and control group on day 7. OTM distance in the rSema3A injection group was significantly reduced compared to that in the control group (n = 5). M1, first molar; M2, second molar. Scale bars = 200 µm. (**c**) Tartrate-resistant acid phosphatase (TRAP) staining and number of TRAP-positive osteoclasts (n = 4). The dotted line shows the compression side of the palatal root. The number of osteoclasts in the rSema3A injection group was significantly reduced compared to that in the control group. Scale bars = 50 µm. (**d**) Double-fluorescence bone labeling and mineral apposition rate of the control and rSema3A injection groups (n = 3). Black square area of schematic diagram is magnified. Alizarin red S bone labels are shown as red lines, and calcein bone labels are shown as green lines. White arrows indicate the mineralized distance after OTM. The mineral apposition rate in the rSema3A injection group was significantly increased compared to the control group. AB, alveolar bone; PDL, periodontal ligament. Scale bars = 50 µm. Data are presented as mean ± SD. *p < 0.05 and **p < 0.01.

Since the effect of rSema3A on osteoblasts and osteoclasts was confirmed, we injected rSema3A into the OTM model and measured the amount of tooth movement, osteoclast number, and bone formation. The tooth impressions showed that the OTM distance in the rSema3A injection group (82.0 ± 9.8 µm, p = 0.000) was significantly reduced compared to that in the control group (124.0 ± 10.2 µm) on day 7 (Fig. 3b). TRAP staining showed the number of osteoclasts in the rSema3A injection group (4.1 ± 0.5 cells/section, p = 0.000) to be significantly reduced compared to that in the control group (7.4 ± 0.4 cells/section) (Fig. 3c). Double-fluorescence bone labeling showed a mineral apposition rate (MAR) of alveolar bone on the tension side. MAR in the rSema3A injection group (10.6 ± 1.5 µm/day, p = 0.006) was significantly increased compared to the control group (4.8 ± 0.4 µm/day) (Fig. 3d).

### *Sema3A* expression after mechanical stress

Previous study^20^ had shown that mechanical stress increased *Sema3A* expression in MC3T3-E1 cells. Therefore, we applied compression and tension stress that mimics OTM to maxillary bone-derived osteoblasts and observe the changes in *Sema3A* mRNA expression.

**Figure 4:**
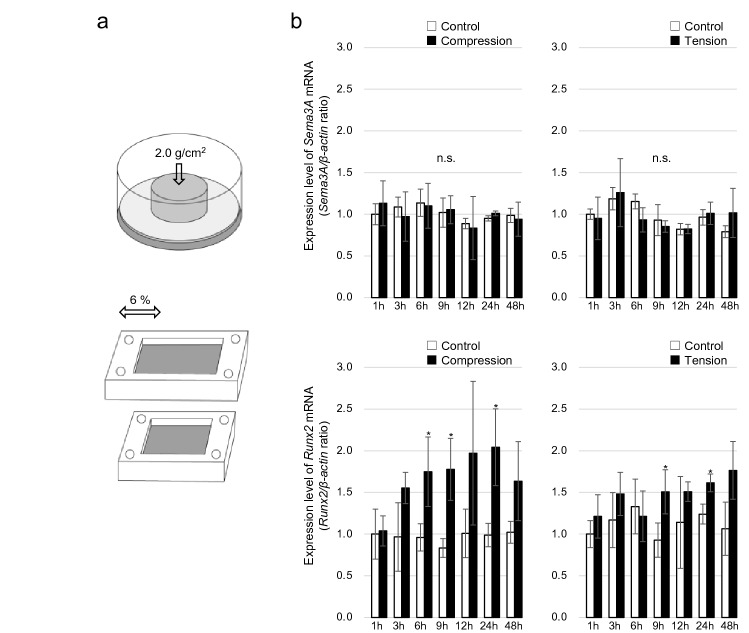
(**a**) The method used to apply compressive force or tension force. Cells were compressed continuously using a glass plate on a 6-well plate or stretched using a stretch chamber. (**b**) *Sema3A* and *Runx2* mRNA quantification by RT-qPCR in osteoblasts after compression and tension stress. The bars represent *Sema3A* and *Runx2* mRNA levels relative to *β-actin* mRNA levels. There were no significant differences in *Sema3A* expression in either mechanical compression or tension stress compared to controls. (n = 3) Data are presented as mean ± SD. *p < 0.05, **p < 0.01, n.s. = no significance.

Osteoblasts were cultured with or without a continuous compressive force (2.0 g/cm^2^) or tension force (6% elongation) for up to 48 h, and the gene expression levels of *Sema3A* and *Runx2* in these cells were determined by RT-qPCR. There were no significant differences in *Sema3A* expression in either mechanical compression or tension stress compared to controls (compression 1 h, p = 0.504; 3 h, p = 0.570; 6 h, p = 0.850; 9 h, p = 0.992; 12 h, p = 0.820 ; 24 h, p = 0.066; 48 h, p = 0.728), (tension 1 h, p = 0.756; 3 h, p = 0.784; 6 h, p = 0.088; 9 h, p = 0.543; 12 h, p = 0.981; 24 h, p = 0.599; 48 h, p = 0.272), and *Runx2* expression was significantly increased under compression stress at 6, 9, and 24 h and tension stress at 9 and 24 h (compression 1 h, p = 0.976; 3 h, p = 0.087; 6 h, p = 0.038; 9 h, p = 0.014; 12 h, p = 0.141; 24 h, p = 0.017; 48 h, p = 0.099), (tension 1 h, p = 0.319; 3 h, p = 0.268; 6 h, p = 0.666; 9 h, p = 0.040; 12 h, p = 0.322; 24 h, p = 0.016; 48 h, p = 0.071) (Fig. 4b).

### *Sema3A* expression after inflammatory cytokine exposure

Since compression and tension stress did not alter osteoblast Sema3A mRNA expression, four cytokines that increase during OTM^16,21–23^ were added to osteoblasts to investigate whether they affect mRNA expression.

**Figure 5:**
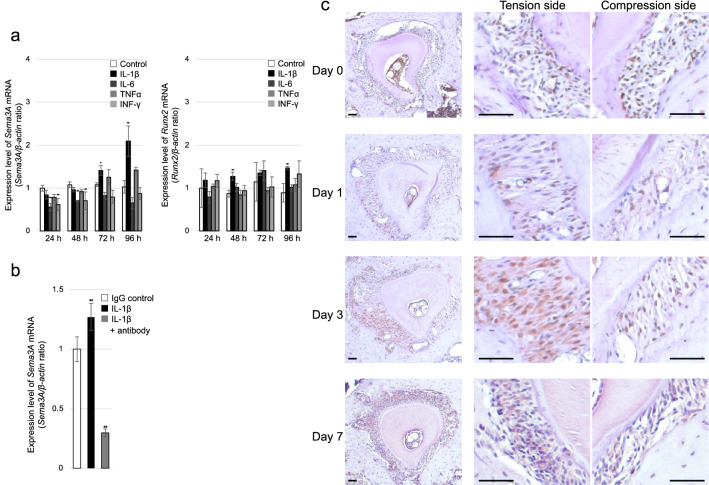
(**a**) *Sema3A* and *Runx2* mRNA quantification by RT-qPCR in osteoblasts after 24, 48, 72, and 96 h in the absence or presence of inflammatory cytokines. *Sema3A* expression was significantly increased at 72 h and 96 h with IL-1β and significantly reduced after 24 h and 48 h with IL-6 and IFN‐γ. The bars represent *Sema3A* and *Runx2* mRNA levels relative to *β-actin* mRNA levels. (n = 3) (**b**) *Sema3A* mRNA quantification by RT-qPCR in osteoblasts treated with IgG control, IL-1β and IL-1β receptor antibody. (n = 5) Data are presented as mean ± SD. *p < 0.05 and **p < 0.01. (**c**) Immunohistochemistry for IL-1β. Horizontal sections at days 0, 1, 3, and 7 after OTM. There were more IL-1β-positive cells in the tension side compared to the compression side on days 1, 3, and 7. Scale bars = 50 µm.

Osteoblasts were cultured with or without IL-1β, IL-6, TNFα, and IFN-γ for up to 96 h, and the gene expression levels of *Sema3A* and *Runx2* were determined by RT-qPCR. *Sema3A* expression was significantly increased at 72 h (p = 0.023) and 96 h (p = 0.000) with IL-1β and significantly reduced after 24 h and 48 h with IL-6 (24 h, p = 0.001; 48 h, p = 0.004) and IFN‐γ (24 h, p = 0.003; 48 h, p = 0.004). *Runx2* expression was significantly increased at 48 h and 96 h with IL-1β (48 h, p = 0.002; 96 h, p = 0.003) (Fig. 5a). *Sema3A* expression was significantly increased with IL-1β (p = 0.000) and significantly reduced with IL-1β receptor antibody (p = 0.000) compared to IgG control (Fig. 5b).

### Expression of IL-1β after OTM

Immunohistochemistry revealed IL-1β-positive cells before and after OTM. On day 0, IL-1β was observed at equal levels in the PDL on the compression and tension sides. There were more IL-1β-positive cells in the tension side compared to the compression side on days 1, 3, and 7 (Fig. 5c).

## Discussion

Although it is known that Sema3A acts on osteoblast and osteoclast differentiation, OTM is explained by the RANK/RANKL coupling mechanism^1,2^. We hypothesized that the OTM mechanism could also be explained by Sema3A. In this experiment, we created a mouse OTM model, and osteoblasts from mouse maxilla and osteoclasts from mouse femur and tibia were used in vitro to explain OTM by Sema3A. The effect of Sema3A in a mouse model of OTM and the inflammatory cytokines that control *Sema3A* expression during OTM were shown. Binding of RANK and RANKL is essential for osteoclast differentiation, function, and survival, and many OTM studies have examined the ratio of RANK and RANKL and their association with OPG, a decoy receptor for RANKL molecules produced by osteoblasts. In the present study, Sema3A was shown to be secreted during OTM and to regulate osteoblasts and osteoclasts.

We focused on osteoblasts and osteoclasts during OTM. Although various cells control OTM, osteoblasts and osteoclasts adhere to the alveolar bone surface and directly resorb and mineralize bone during OTM^3,4^. Immunofluorescent staining of Sema3A on day 3 (Fig. 1c) showed strong fluorescence around Sp7-positive cells and structures that appeared to be blood vessels in the periodontal ligament. However, the fluorescence of this structure was also observed on Day 0, suggesting that it is expressed regardless of OTM. Therefore, we considered that Sema3A, which expression increased on the tension side during OTM, was mainly secreted by osteoblasts. RT-qPCR also confirmed the expression of *Sema3A* mRNA in osteoblasts from the maxilla of mice. However, Sema3A is a secreted protein that is secreted by human PDL fibroblasts^13^ and other tissues. Therefore, further experiments are needed to determine which cells produce Sema3A in OTM.

Sema3A was observed in the PDL on day 0 of OTM. Sema3A is thought to play an important role during the bone formation phase by preventing osteoclasts from approaching new bone formation sites by osteoblasts^7^. The alveolar bone around the root of the tooth is constantly remodeled^21^, and the osteoblasts on the surface of the alveolar bone may also secrete Sema3A during remodeling to keep osteoclasts away from the bone formation site. Sema3A expression was higher on the tension side than on the compression side on day 1 and 3. The number of osteoclasts on day 7 was lower on the tension side than on the compression side. On the compression side of the root, the decrease in Sema3A inhibited osteoblast differentiation and promoted osteoclast differentiation, creating an environment for bone resorption. On the tension side, the increase in Sema3A promoted osteoblast differentiation and inhibited osteoclast differentiation, creating an environment for bone formation.

To explain the difference in Sema3A expression between the compression and tension sides, we performed mechanical stress on osteoblasts. It has been found that mechanical stress changes *Sema3A* expression in MC3T3-E1 cells^20^, and we examined the effects of mechanical stress on osteoblasts during compression and tension that mimics OTM. In this study, we applied a compression force of 2.0 g/cm^2^ or 6% tension force to osteoblasts for 1–48 h. The application of compressive force to osteoblasts is based on previous studies^15,24–26^. A previous study found that 16 × g and 64 × g of centrifugal force increased *Sema3A* mRNA expression in MC3T3-E1 cells^20^. A previous study used centrifugal force to investigate postmenopausal osteoporosis; however, our compression and tension experiment that mimicked OTM showed no change in *Sema3A* mRNA expression. In a previous study, compression (30 g/cm^2^) or tension (2.5%) stress were applied to human alveolar bone osteoblasts, no change in Sema3A mRNA was observed in both. This is the same result as our experiment. On the other hand, when the same mechanical stress was applied to primary human periodontal fibroblasts, the expression levels of mRNA and protein were increased by tension and decreased by compression stress^27^. These results suggest that Sema3A in periodontal ligament during OTM may also be secreted by periodontal fibroblasts.

IL-1β stimulation increased *Sema3A* mRNA expression by RT-qPCR, and more IL-1β was observed on the tension side of OTM on days 1, 3, and 7 compared to the compression side on immunohistochemistry. It has been reported that IL-1β increases when a tension force is applied to the human PDL^28,29^, and IL-1β is increased in gingival crevicular fluid from both the compression and tension sides during OTM, but more so on the tension side^22^. Our immunohistochemistry results were consistent with these results. However, our experiments did not reveal how IL-1β regulates *Sema3A* expression. Further experiments are needed to explain the mechanism of *Sema3A* upregulation by IL-1β. In addition, it was also shown that IL-6 and IFN‐γ reduced *Sema3A* mRNA expression in osteoblasts. These inflammatory cytokines also regulate *Sema3A* expression and may be involved in OTM. In summary, these results indicate that, on the tension side, IL-1β stimulates osteoblasts, leading to bone formation and inhibiting osteoclastogenesis by increasing Sema3A secretion.

In conclusion, Sema3A secreted by osteoblasts controls alveolar bone remodeling during OTM. It inhibits bone resorption on the compression side by inhibiting osteoclastogenesis and promotes bone mineralization on the tension side by promoting osteoblast calcification. Sema3A secretion from osteoblasts during OTM was upregulated by IL-1β in the PDL rather than by mechanical stress. This experiment helps us to explain one of the mechanisms of alveolar bone remodeling during OTM.

## Methods

### Orthodontic tooth movement model (OTM model)

All animal experiments were approved by the Tokyo Dental College Animal Research Ethics Committee (No. 203104) and performed in accordance with the relevant guidelines and regulations. The study protocol also complied with the ARRIVE Guidelines. Sixty 8-week-old C56BL/6 J wild-type male mice were used for in vivo. After intraperitoneal administration of a combination anesthetic [medetomidine hydrochloride, 0.3 mg/kg (Domitol; Nippon Zenyaku Kogyo, Koriyama, Japan); midazolam, 4.0 mg/kg (Sandoz; Sandoz, Holzkirchen, Germany); and butorphanol, 5.0 mg/kg (Vetorphale; Meiji Seika Pharma, Tokyo, Japan)], 10 gf of nickel-titanium (Ni–Ti) closed-coil spring (TOMY, Tokyo, Japan) was attached to the maxillary incisor and the maxillary left first molar (Fig. 1a). After days 0 (control), 1, 3, and 7, the mice were sacrificed and analyzed.

### Tissue preparation

Anesthetized mice were sacrificed and fixed with 4% paraformaldehyde. The maxilla was decalcified in ethylenediaminetetraacetic acid (EDTA) for 10 days at 4 °C. Samples were embedded in paraffin and cut into sections 4-µm thick. Horizontal sections of the first molar palatal root region were prepared. Three levels (40, 80, and 120 µm) from the bifurcation area were evaluated for each sample.

### Measurement of osteoclast number

To estimate the number of osteoclasts during OTM, tartrate-resistant acid phosphatase (TRAP) staining was performed. Sections were incubated in acetate buffer containing naphthol AS-BI phosphoric acid solution, Fast Garnet GBC base solution, sodium solution, and tartrate solution (Sigma-Aldrich, St. Louis, MO), and then counterstained with hematoxylin. The number of TRAP-positive multinucleated cells containing more than three nuclei was determined by microscopy. Three sections of the mesial side of the palatal root at 40, 80, and 120 µm from the bifurcation in each mouse were evaluated and four biological replicates were analyzed, and the number of osteoclasts was expressed as the average of these three sections. UPM Axio Phot2 (Carl-Zeiss, Jena, Germany) was used for TRAP staining.

### Immunofluorescent staining of Sema3A

The distribution of Sema3A and osteoblasts in OTM was observed by staining for Sema3A and Sp7, an osteoblast marker. The sections were incubated with Alexa Fluor 488-conjugated Sema3A mouse antibody (sc-74554 AF488, Santa Cruz Biotechnology, Santa Cruz, CA), Sp7 rabbit antibody (ab209484, Abcam) or isotype control antibody (Biorbyt), followed by species-matched Alexa Fluor 546-conjugated secondary antibody (A10040, Invitrogen). Nuclei were stained with Hoechst 33342 (Dojindo). Staining of mouse kidney and mouse nasal bone samples were performed as positive control (Supplementary Figure S1). Sections were observed using an LSM 880 microscope (Carl-Zeiss) and quantification of the staining density was analyzed using Image J software (version 1.53 k, https://imagej.nih.gov/ij/; National Institutes of Health, Bethesda, MD). Sema3A fluorescence area within 50 µm from the alveolar bone surface was analyzed and expressed as a percentage of positive Sema3A staining. Five biological replicates were analyzed.

### Immunohistochemistry of IL-1β

Immunohistochemical staining was performed to detect IL-1β protein during OTM. After blocking the endogenous peroxidase activity in methanol/H_2_O_2_ for 30 min, the sections were preincubated with 1% bovine serum albumin (Sigma-Aldrich) for 10 min to avoid non-specific background staining. Slices were then incubated with an avidin/biotin blocking reagent (BioCare Medical, Concord, CA) to block endogenous avidin and biotin binding sites. The sections were stained with IL-1β antibody (MAB401R, R&D Systems, Minneapolis, MN, dilution 1:200) or IgG1 Isotype Control at 4 °C overnight. Slices were incubated with biotinylated secondary antibodies (Iwai Chemicals, Tokyo, Japan) at 4 °C overnight. After incubation, streptavidin–horseradish peroxidase (BioLegend, San Diego, CA) was applied for 60 min. The DAB Substrate Kit (Vector Laboratories, Burlingame, CA) was used for color development according to the manufacturer’s instructions. The sections were counterstained with hematoxylin. Staining of mouse skin sample 48 h after LPS injection was performed as positive control (Supplementary Figure S2). Sections 40 µm from the bifurcation were observed for immunohistochemical staining using UPM Axio Phot2 (Carl-Zeiss).

### Primary mouse maxillary osteoblast culture

The maxillary bone of C56BL/6J mice was obtained, and the first molar was extracted using an injection needle under a microscope. After removal of the soft tissue, the maxillary bone was treated with PBS containing 0.1% collagenase (Wako Pure Chemical, Osaka, Japan) and 0.2% Disperse II (Godo Shusei, Chiba, Japan). The digestion procedure was repeated three times, and the cells collected here were cultured as osteoblasts. The cells were suspended in α-MEM culture medium containing penicillin, streptomycin, and 10% fetal bovine serum. When the cells were 80% confluent, they were resuspended in a 6-well plate or a stretch chamber (1 × 10^4^ cells/cm^2^). To initiate osteoblastic differentiation, confluent cells were treated with osteogenic culture medium (OCM; 100 mM ascorbic acid, 5 mM β-glycerophosphate, and 10 nM dexamethasone). After 7 days, mechanical stress and inflammatory cytokine exposure [100 ng/mL interleukin-1β (IL-1β, R&D Systems), interleukin-6 (IL-6, R&D Systems), tumor necrosis factor α (TNFα, R&D Systems), and interferon-γ (IFN‐γ, R&D Systems) for 24, 48, 72, and 96 h] were performed. To confirm the effect of IL-1β, 100 ng/mL IL-1β (R&D Systems) and 50 ng/mL IL-1β receptor antibody (Mybiosource, San Diego, CA) or 50 ng/mL IgG control (MAB005, R&D Systems) exposure were performed. The cells were treated with IL-1β receptor antibody or IgG control on day 7, and IL-1β on day 8 for 96 h. The cells were maintained at 37 °C in an atmosphere containing 5% CO_2_, and the medium was changed every 3 days.

### Measurement of alkaline phosphatase (ALP) for osteoblast activity in vitro

To measure ALP activity, ALP assay and ALP staining were performed. Osteoblasts were cultured in OCM with or without rSema3A (10, 50 ng/mL) on a 48-well plate, and ALP was measured on day 7. ALP activity was assayed using LabAssay ALP (Wako). The release of *p*-nitrophenol from *p*-nitrophenylphosphate was measured by optical density at 405 nm using a Synergy H1 plate reader (Bio Tek, Winooski, VT). ALP staining was performed using a TRAP/ALP kit (Wako) according to the manufacturer’s instructions. After fixing with 4% paraformaldehyde, ALP was stained with a pre-mixed substrate mixture for 30 min at 37 °C. Control cells were cultured in OCM. Three technical replicates were analyzed.

### Measurement of mineralized nodule formation

To measure extracellular calcification, alizarin red S staining was performed. Osteoblasts were cultured in OCM with or without rSema3A (10, 50 ng/mL) on a 48-well plate, and mineralized nodules were confirmed by alizarin red S staining (Sigma-Aldrich) at 21 days of culture. Quantification of the staining density was performed using ES-10000G (Seiko Epson, Nagano, Japan) and analyzed using Image J software (National Institutes of Health, Bethesda, MD). Control cells were cultured in OCM. Three technical replicates were analyzed.

### Osteoclast culture

Mononuclear bone marrow-derived monocyte/macrophage precursor cells (BMMs) were isolated from the femur and tibia of 8-week-old C57BL/6 J male mice by the density gradient centrifugation method using Histopaque 1083 (Sigma-Aldrich). The resulting BMMs were seeded into 48-well plates (1 × 10^5^ cells/cm^2^) in α-MEM media supplemented with 10% fetal bovine serum and 50 ng/mL macrophage colony-stimulating factor (M-CSF; BioLegend). After incubation for 2 days, the medium was replaced with fresh medium containing M-CSF (50 ng/mL) and murine soluble RANKL (100 ng/mL; BioLegend) with or without rSema3A (10, 50 ng/mL). The cells were maintained at 37 °C in an atmosphere containing 5% CO_2_, and the medium was changed every 2 days.

### TRAP staining of BMMs

TRAP staining of BMMs was performed after fixing with 4% paraformaldehyde using a TRAP/ALP staining kit (Wako) according to the manufacturer’s instructions at 6 days after RANKL stimulation. The number of TRAP-positive multinucleated cells containing more than three nuclei was counted by microscopy. Control cells were cultured without rSema3A. Three technical replicates were analyzed.

### Local injection of Sema3A recombinant protein

Injection of recombinant Sema3A protein (rSema3A) was performed to assess Sema3A during OTM. Mouse rSema3A (5 µg, 5926-S3, R&D Systems) or phosphate-buffered saline (PBS) was injected locally into the palatal and buccal gingiva of the left first molar using a microliter syringe (Hamilton, Reno, NV) with a 30-G needle 1 day before Ni–Ti spring attachment.

### Measurement of OTM

The amount of movement was measured as previously described^30^. The distance of tooth movement was measured on day 7 after the attachment of the Ni–Ti coil spring. Impressions of the teeth were obtained with individual trays (Tray resin; Shofu, Kyoto, Japan) and hydrophilic vinyl polysiloxane dental impression material (Exafine, GC Co., Tokyo, Japan). The impression was cut on the line connecting the middle of the distal fissure in the first molar and the middle of the mesial fissure in the second molar. The distance of tooth movement was measured by the thickness of the dental impression material between the first and second molars at the level of the contact point. The impression was observed using a Stemi 508 microscope (Carl-Zeiss). Five biological replicates were analyzed.

### Double-fluorescence bone labeling

Calcein (20 mg/kg; Dojindo) was intraperitoneally injected 24 h before Ni–Ti coil spring attachment, and alizarin red S (40 mg/kg; Sigma-Aldrich) was injected 24 h before sacrifice (Fig. 3a). OTM proceeded for 7 days, after which the maxilla was collected and fixed with 4% paraformaldehyde. The samples were dehydrated through an ethanol gradient (70–100%) and embedded in Rigolac (Showa Denko, Tokyo, Japan). Samples were cut into 200-µm-thick sections with an Isomet slow-speed saw and an ultrathin diamond wafering blade (Buehler, Coventry, UK). An LSM 880 microscope (Carl-Zeiss) was used to observe the fluorescence. To compare the MAR between the control and rSema3A injection groups, the distance between the calcein and alizarin red S signals was measured in the distal segment of the palatal root. The excitation/emission wavelengths used for fluorescence were 488/517 nm (calcein) and 543/617 nm (alizarin red S). Three biological replicates were analyzed.

### Mechanical stress for in vitro experiments

#### Application of compressive force

Osteoblasts were cultured in 6-well plates with or without continuous compressive force (2.0 g/cm^2^) for 1, 3, 6, 9, 12, 24, and 48 h. Thin, round glass plates were placed over the cells, and the compressive force was adjusted by placing a weight on the glass plates. A total of 82.6% of the cells in the culture dish were covered with a glass plate (Fig. 4a).

#### Application of tension force

ShellPa Pro (Menicon Life Science, Nagoya, Japan) was used to generate cyclic tensile strain in osteoblasts. According to the manufacturer’s instructions, the cells were subjected to mechanical strain of 6% elongation at 6 cycles/min for 1, 3, 6, 9, 12, 24, and 48 h (Fig. 4a). Control cells were cultured on the same plates and kept in the same incubator without mechanical strain.

### Reverse transcription quantitative real-time PCR (RT-qPCR) analysis

Total RNA was isolated with TriZol (Invitrogen), and the isolated mRNA was evaluated in quantity and quality using a Nanodrop ND-100 spectrophotometer (Thermo Scientific, Waltham, MA). mRNA was converted to cDNA using ReverTra Ace qPCR RT Master Mix with gDNA Remover (Toyobo, Osaka, Japan). For RT-qPCR, a reaction mixture was prepared with Thunderbird SYBR qPCR Mix (Toyobo), paired primers, and a defined amount of template cDNA. RT-qPCR reactions were carried out using the following primer sets for *β-actin*, *Sema3A* and *Runx2* : *β-actin*: 5’-CGGTTCCGATGCCCTGAGGCTCTT-3’ and 5’-CGTCACACTTCATGATGGAATTG A-3’; *Sema3A*: 5’-TGGGCTGGTTCACTGGGATTGC-3’ and 5’-CTGGAGCTGTTGGCCAAGCCAT-3’; *Runx2*: 5’-AATTAACGCCAGTCGGAGCA-3’ and 5’-CACTTCTCGGTCTGACGACG-3’. RT-qPCR was performed using a 7500 Fast Real-Time PCR system (Applied Biosystems, Foster City, CA) and 7500 Fast System SDS Software (version 1.5.1, Applied Biosystems). Initial denaturation was induced at 95 °C for 24 s, followed by 40 cycles of denaturation at 95 °C for 3 s, annealing at 60 °C for 5 s, and elongation at 72 °C for 45 s. The relative expression ratio of markers was calculated based on the ddCt comparative threshold cycle (CT) method. The calculated values were normalized against an internal control (*β-actin*). Five technical replicates were analyzed for RT-qPCR.

### Statistical analysis

Results are expressed as mean ± SD. Statistical analyses were performed using SPSS Statistics version 24 (IBM, Armonk, NY). All data were analyzed using Student’s *t*-test (Figs. 1c, 3, 4b) or one-way analysis of variance (ANOVA) followed by a post hoc Tukey’s (Figs. 1b, 2) or Dunnett’s (Fig. 5) test. A *p*-value of < 0.05 was considered statistically significant.

## Supplementary Information

Supplementary Figures.

## Data Availability

All data generated or analyzed during this study are included in this published article.
