# Supplementary material for: Semaphorin 3A regulates alveolar bone remodeling on orthodontic tooth movement
Source: Sci Rep. 2022 Jun 2;12:9243. doi: 10.1038/s41598-022-13217-x (PMC9163121; doi:10.1038/s41598-022-13217-x)
Supplement: Supplementary file 1 — Supplementary Figures. [file 41598_2022_13217_MOESM1_ESM.docx]

Supplementary Figure S1

Positive and negative control of Sema3A and Sp7 immunofluorescent staining.

Mouse kidney and nasal bone samples were stained.

Scale bars = 100 µm.

Supplementary Figure S2

Positive and negative control of IL-1β DAB immunohistochemistry.

Mouse skin samples were collected 48 hours after LPS stimulation for IL-1β induction.

Scale bars = 100 µm.
